# Toward a Consensus Model of Cognitive–Reading Achievement Relations Using Meta-Structural Equation Modeling

**DOI:** 10.3390/jintelligence13080104

**Published:** 2025-08-16

**Authors:** Daniel B. Hajovsky, Christopher R. Niileksela, Dawn P. Flanagan, Vincent C. Alfonso, William Joel Schneider, Jacob Robbins

**Affiliations:** 1 Department of Educational Psychology, Texas A&M University, College Station, TX 77845, USA; jarobbins93@tamu.edu; 2 Department of Educational Psychology, University of Kansas, Lawrence, KS 66045, USA; chrisn@ku.edu; 3 Department of Psychology, St. John’s University, Queens, NY 11439, USA; flanagad@stjohns.edu; 4 College for Education and Engaged Learning, Montclair State University, Montclair, NJ 07043, USA; alfonsov@montclair.edu; 5 Psychological Studies in Education, Temple University, Philadelphia, PA 19122, USA; schneider@temple.edu

**Keywords:** cognitive abilities, reading skills, cognitive–achievement relations, CHC theory, meta-structural equation modeling

## Abstract

Cognitive tests measure psychological constructs that predict the development of academic skills. Research on cognitive–reading achievement relations has primarily been completed with single-test batteries and samples, resulting in inconsistencies across studies. The current study developed a consensus model of cognitive–reading achievement relations using meta-structural equation modeling (meta-SEM) through a cross-sectional analysis of subtest correlations from English-language norm-referenced tests. The full dataset used for this study included 49,959 correlations across 599 distinct correlation matrices. These included correlations among 1112 subtests extracted from 137 different cognitive and achievement test batteries. The meta-SEM approach allowed for increased sampling of cognitive and academic reading skills measured by various test batteries to better inform the validity of construct relations. The findings were generally consistent with previous research, suggesting that cognitive abilities are important predictors of reading skills and generalize across different test batteries and samples. The findings are also consistent with integrated cognitive–reading models and have implications for assessment and intervention frameworks.

## 1. Introduction

Reading proficiency is an essential skill within and beyond academic settings. Within school contexts, skills acquired in kindergarten, like phonemic awareness, predict later academic performance, such as better Reading Comprehension in third grade (Snowling et al. 2022, p. 345). Additionally, when early elementary school students do not develop sufficient reading skills, they face heightened risks at the high school level, such as lower overall grades and a higher likelihood of dropping out (e.g., Lesnick et al. 2010). Beyond formal schooling, higher literacy is associated with increased job opportunities and earnings, as well as greater community engagement and parenting practices (e.g., Kutner et al. 2007; Smart et al. 2017). Conversely, lower literacy is linked to poor understanding of medical information (which may precipitate ill health) and a greater likelihood of delinquency (e.g., Ritchie and Bates 2013). Thus, fostering reading development supports proximal success and distal well-being. For professionals who seek to cultivate these outcomes (i.e., teachers, interventionists, clinicians), knowing the cognitive components of reading can be particularly useful (Tunmer and Hoover 2019).

Accumulating evidence suggests that general intelligence and specific cognitive abilities predict reading skills, a line of research referred to here as cognitive–reading relations. However, it remains unclear to what extent cognitive–reading relations are generalizable across different measures rather than being test-specific (American Educational Research Association et al. 2014; Messick 1995). Although prior research supports the notion that cognitive abilities are measured consistently across cognitive ability batteries (Caemmerer et al. 2020; Niileksela and Reynolds 2019; Reynolds et al. 2013), there has been no large-scale systematic study that combines relations among cognitive and reading achievement test batteries. The aim of this study is to analyze multiple cognitive and reading tests simultaneously using meta-structural equation modeling (meta-SEM) in a cross-sectional analysis based on English-language data to further examine integrated theoretical frameworks of reading and cognitive abilities. In doing so, the findings from the current study help inform a consensus assessment model, which, in turn, may be useful in guiding instruction and intervention (Tunmer and Hoover 2019).

### 1.1. Theoretical Frameworks of Reading

Traditionally, the Simple View of Reading (SVR) asserted that reading is the product of word reading and language comprehension (Gough and Tunmer 1986). Across the ocean of reading research conducted since SVR’s inception, several updated models have surfaced. Two of these are the Comprehensive Model of Discourse Comprehension (CMDC; Van Den Broek and Kendeou 2022) and the Direct and Indirect Effects Model of Reading (DIER; Kim 2023). These models build on SVR by accounting for dynamic individual and contextual factors that transcend word reading and language comprehension to provide updated perspectives on the structure and process of reading.

Central to CMDC is the construction of a mental representation, referred to as a situation model, which emerges dynamically as readers integrate new textual information with prior knowledge through passive (automatic) and strategic (deliberate) inferential processes. The model emphasizes that comprehension is a cyclical process where each segment of text activates relevant knowledge and prior textual content, facilitating the establishment of semantic relations—primarily referential and causal—that underpin coherence. Critical to this integration process are the readers’ standards of coherence, or the criteria guiding their comprehension efforts, determining whether passive processes suffice or reader-initiated actions (e.g., rereading, reflecting, evaluating) are required. DIER builds on CMDC by identifying the component skills of Reading Comprehension and proposes a hierarchical structure. Therein, phonological, orthographic, and morphological awareness contribute to word reading; vocabulary, grammatical knowledge, and background knowledge contribute to Listening Comprehension; and domain general cognitive abilities (e.g., working memory) feed into each. Although the overall model is grounded in word reading and Listening Comprehension, Reading Fluency serves as a bridge, linking word reading and Listening Comprehension to Reading Comprehension (Kim 2023).

In line with DIER and CMDC, there is a growing impetus for integrated cognitive–reading relations models that posit cognitive abilities as influencing advanced reading skills through basic reading skills (e.g., Feraco et al. 2023; Hajovsky et al. 2014; Niileksela et al. 2016). For example, general intelligence and broad cognitive abilities (e.g., Comprehension-Knowledge, Working Memory) influence Reading Comprehension through basic reading skills (Floyd et al. 2012; Hajovsky et al. 2014). To derive such insights, cognitive–reading relations research is often guided by the Cattel–Horn–Carroll (CHC) theory, discussed below.

### 1.2. Cattell–Horn–Carroll (CHC) Theory of Intelligence

The Cattell–Horn–Carroll (CHC) theory is a hierarchical taxonomy of human cognitive abilities widely used in assessment research, development, and interpretation (Schneider and McGrew 2018). At its apex (Stratum III), CHC theory posits a single general factor, psychometric g, representing a broad measure of general intelligence. Stratum II comprises 17 broad cognitive abilities (e.g., Fluid Reasoning, Processing Speed, Comprehension-Knowledge) that share underlying cognitive processes and reflect *g* to varying degrees, often represented by composite scores on cognitive test batteries. At the most specific level (Stratum I), CHC theory identifies over 80 narrow abilities such as Phonetic Coding, Lexical Knowledge, Working Memory, and General Sequential Reasoning, each assessed by individual subtests. CHC theory provides a strong theoretical and empirical framework to guide research on cognitive–achievement relations.

### 1.3. Cognitive–Achievement Relations in Reading

Cognitive–reading relations research investigates basic reading skills, Reading Fluency, and Reading Comprehension. Basic reading encompasses decoding and word recognition, both of which are associated with multiple broad cognitive abilities (Floyd et al. 2012; Hajovsky et al. 2014; Niileksela et al. 2016). Specifically, Comprehension-Knowledge, Short-Term Memory, and Processing Speed have demonstrated relationships with basic reading skills among school-age children. One of the most robust cognitive–reading relations observed is the one between Comprehension-Knowledge and Reading Comprehension (Caemmerer et al. 2018; Floyd et al. 2012; Hajovsky et al. 2014; Keith 1999; Niileksela et al. 2016). Additionally, Short-Term Memory, Processing Speed, and Fluid Reasoning appear important for Reading Comprehension, albeit with less consistent support (Cain et al. 2004; Cormier et al. 2017; Floyd et al. 2012; Hajovsky et al. 2014; Niileksela et al. 2016). For Reading Fluency, growing evidence suggests that Comprehension-Knowledge, Processing Speed, and Fluid Reasoning are predictors (Caemmerer et al. 2018; Cormier et al. 2017; Niileksela et al. 2016).

The pattern and strength of the abovementioned relationships vary depending on test batteries (cf. Caemmerer et al. 2018; Cormier et al. 2017; Evans et al. 2002; Floyd et al. 2007; Hajovsky et al. 2014; Niileksela et al. 2016). For instance, Hajovsky et al. (2014) reported an atypical finding indicating a relationship between Visual Processing and Reading Comprehension for younger children assessed using the Kaufman Assessment Battery for Children—Second Edition (KABC-II, Kaufman and Kaufman 2004). Such mixed findings have, in part, contributed to the uncertainty surrounding the use of cognitive assessments for identifying Specific Learning Disabilities (SLDs), such as dyslexia (Grigorenko et al. 2020; Machek and Nelson 2010). Thus, gaining more comprehensive insights using cross-battery research is warranted.

### 1.4. Cross-Battery Meta-Analytic Research

Cross-battery meta-analyses allow for a comprehensive examination of intelligence, the broad and narrow abilities therein, and basic and advanced academic skills (e.g., Johnson et al. 2010; Phelps et al. 2005; Sanders et al. 2007). In addition to the benefits of a typical meta-analysis, where different samples, sample sizes, and measures can be integrated, taking a cross-battery approach provides unique advantages, particularly in terms of statistical power. Intelligence and academic achievement test batteries typically include two to three measures of a broad cognitive ability (e.g., Working Memory) or academic skill (e.g., Reading Comprehension) within a single battery. Using a cross-battery method, simultaneous analysis of multiple batteries can be conducted, allowing for a more comprehensive sampling of subtests for each ability or skill.

In terms of previous work, two comprehensive meta-analyses have been conducted. Bryan and Mayer (2020) examined relations among broad abilities in 61 studies conducted since 1960. Their meta-analysis focused on relations across composite scores and latent variables. They found that for reading and writing, there was a correlation of *r* = 0.85 with Comprehension-Knowledge and a correlation of *r* = 0.62 with Long-Term Storage. Smaller effects included Short-Term Memory (*r* = 0.45), Auditory Processing (*r* = 0.37), and Processing Speed (*r* = 0.25).

Zaboski et al. (2018) analyzed 25 studies from 1988 to 2009. They found that general intelligence (*g*) demonstrated the strongest and most consistent relationships with basic reading and Reading Comprehension. Specifically, *g* accounted for approximately 54% of variance overall, with correlations averaging *r* = 0.74 for basic reading and *r* = 0.76 for comprehension. Among the broad cognitive abilities, only Comprehension-Knowledge explained consistent variance, yielding correlations of *r* = 0.45 for basic reading and Reading Comprehension. Additional broad abilities explained relatively smaller proportions of variance. Auditory Processing and Short-Term Memory were the next highest coefficients for basic reading (*r* = 0.34 and *r* = 0.28, respectively), whereas Long-Term Storage and Retrieval (*r* = 0.24), Auditory Processing (*r* = 0.18), and Short-Term Memory (*r* = 0.16) were linked to Reading Comprehension.

There are some substantial differences in the relationships found between the Bryan and Mayer (2020) and Zaboski et al. (2018) studies. The primary difference appears to be that the Zaboski et al. study uses relationships between broad abilities and academic skills after removing relationships due to *g* and other broad abilities, whereas the Bryan and Mayer study estimates the correlations without removing the effects of *g*. The current study is designed in a way that allows for an examination of cognitive–reading relations by estimating the correlations without removing the effects of *g* and then using those correlations in a model that can estimate the effects of *g* and broad abilities on reading skills.

There is a gap in the literature concerning the empirical relations between cognitive ability and academic achievement variables when analyzed using data from multiple intelligence and achievement test batteries simultaneously. Such an analysis would allow for stronger inferences of cognitive–reading relations that generalize across test batteries. Additionally, this empirical research provides a more suitable framework for estimating the degree to which *g* and broad abilities contribute to an individual’s academic difficulties. Furthermore, although ample evidence suggests there are specific cognitive and achievement relations that vary in magnitude across independent studies of cognitive and achievement relations (e.g., Benson et al. 2016; Caemmerer et al. 2018; Hajovsky et al. 2014; Niileksela et al. 2016; Zaboski et al. 2018), these relations have not been established within a cross-battery confirmatory factor analysis model to determine whether relations are generalizable.

### 1.5. The Current Study

Previous work has not examined cognitive–achievement relations across multiple test batteries simultaneously. It is unclear whether cognitive–achievement relations from previous studies generalize across test batteries and are not test-specific. The current study examines integrated models of cognitive–reading achievement relations across multiple test batteries using meta-SEM (Jak et al. 2021). Normative and special validity samples of multiple standardized cognitive and achievement test batteries are used. Meta-SEM allows for an increased sampling of cognitive abilities and academic skills measured by various batteries, allowing for clearer inferences about construct-specific relations that may generalize across test batteries. This study expands the literature on cognitive–achievement relations by investigating how cognitive abilities and basic reading skills influence advanced reading skills across various test batteries. Based on previous research and theoretical expectations, it is predicted that some specific cognitive abilities (e.g., Comprehension-Knowledge) will have consistent influences on reading skills, and basic reading skills will influence more advanced reading skills across various test batteries.

## 2. Methods

### 2.1. Identification of Published Tests and Manuals

The analysis presented in this study is part of a larger project that aims to identify all published standardized, norm-referenced tests of cognitive abilities and academic achievement and extract the correlation matrices included in their technical manuals. Current and previous versions of tests were included in the full database of correlation matrices, and tests that continue to be developed and revised will be added to the database as part of this larger project. Test manuals that were available from university-based clinics and test libraries were obtained and reviewed for correlation matrices at the subtest level. For this study, measures from 137 different cognitive and achievement test batteries were included. From those test batteries, 599 different correlation matrices were extracted from the test manuals. Those correlation matrices included 1112 subtests. The full list of test batteries, correlation matrices taken from the technical manuals, subtest categorizations, and correlations are located in Supplemental Tables S1–S4, respectively. With the current data, a two-stage meta-analytic structural equation model methodology was used, where the first stage involved using the correlations from the input data to estimate the meta-analytic correlations among all the broad cognitive abilities and reading skills. These were used to create a meta-analytic correlation matrix, which was then used in the second stage to examine relationships between cognitive abilities and reading skills.

### 2.2. Input Data

Correlation matrices that included subtest-level correlations were used for input data for this study. Inclusion criteria for the correlation matrices were as follows: (1) there had to be correlations between subtests only (i.e., not subtests and composite scores, or not only composite scores), and (2) the correlation matrix had to be for the normative sample or a concurrent validity sample. All correlation matrices for the normative samples and concurrent validity samples from the technical manuals were included if they provided subtest-level correlations. Concurrent validity samples were included so cross-battery correlations could also be included, allowing for a broader coverage of correlations across different cognitive and achievement constructs. If a concurrent validity sample included correlations between two versions of the same test (e.g., between the Wechsler Adult Intelligence Scale—Fourth Edition [WAIS-IV] and Wechsler Adult Intelligence Scale—Fifth Edition [WAIS-5]), correlations between the same subtest on different versions of the tests (e.g., similarities from the WAIS-IV with similarities from the WAIS-5) were not included so the within-ability meta-analytic correlations were only between different subtests that measure the same cognitive abilities or academic skills (i.e., the same subtests across test battery revisions would likely include systematic variance due to methodological and/or item overlap). Correlations among subtests for clinical samples that may have been included in the technical manuals were not used for this study to reduce any differences in relationships that may be due to specific clinical diagnoses (e.g., intellectual disability, language disorder). Some technical manuals included a correlation matrix for the full normative sample as well as correlation matrices for different age or grade levels. If a manual included correlation matrices for different age or grade groups, these matrices were used rather than a single correlation matrix for the entire normative sample.

For each correlation matrix, the subtest names, correlation coefficients, and sample size were recorded. All correlation matrices were entered into a spreadsheet, and then all elements of the correlation matrices were extracted and put into a single database that included the data sheet name (e.g., WISC-V Age 6 years 0 months to 6 years 11 months), the subtest names included in each correlation, the broad CHC ability categorization for both subtests, the narrow CHC ability categorization for both subtests, the sample size for the correlation matrix, and the correlation coefficient. For correlation matrices with different sample sizes for different pairs of variables, the actual sample size used for that pair was recorded when available.

### 2.3. Broad and Narrow CHC Ability Categorization of Subtests

All subtests obtained through test manuals were categorized using broad and narrow ability classifications from the current CHC taxonomy (Schneider and McGrew 2018). Categorization was completed through expert consensus, based on years of research and work related to categorizing and recategorizing tests, informed by updated research (e.g., Flanagan et al. 2025; LaForte et al. 2025, pp. 214–18). The categorizations were primarily completed by the third (DF) and fourth authors (VA), with the fifth author (WS) also contributing to clarifying the classifications based on subtest demands, alignment with CHC theory, and extant research on different test batteries.

Tests categorized under eight broad CHC abilities and five narrow reading skills were used in this study. The eight broad CHC abilities included Comprehension-Knowledge (Gc), the breadth and depth of acquired knowledge, skills, and abilities which includes vocabulary, general information, and cultural knowledge accumulated through experience and education; Fluid Reasoning (Gf), the ability to think logically and solve novel problems independently of acquired knowledge which involves reasoning, pattern recognition, and abstract thinking with unfamiliar material; Visual Processing (Gv), the ability to perceive, analyze, synthesize, and think with visual patterns which includes spatial visualization, mental rotation, and processing visual spatial information; Auditory Processing (Ga), the ability to perceive, analyze, and synthesize auditory information which includes discriminating sounds, analyzing auditory patterns, and processing temporal auditory information; Long-Term Storage, also called Learning Efficiency (Gl), the ability to learn, store, and consolidate new information over periods of time measured in minutes, hours, or days and includes the efficiency with which new information can be encoded into long-term stores for later access; Retrieval Fluency (Gr), the ability to fluently and rapidly retrieve information from long-term stores, which involves the speed and ease with which previously learned information can be accessed and recalled, particularly in the tasks that require the quick retrieval of well-learned material; Short-Term Working Memory (Gwm), the ability to temporarily store and manipulate information in immediate awareness while performing cognitive operations on that information; and Processing Speed (Gs), the ability to perform automatic cognitive tasks quickly and efficiently particularly under pressure to maintain focused attention and concentration. The narrow reading skills were Lexical Decoding (LD), direct visual recognition of whole words or meaningful word parts stored in long-term memory that involves instantly recognizing familiar words as complete visual patterns (sometimes called sight word vocabulary); Phonological Decoding (PD), the systematic translation of letters or letter patterns into their corresponding sounds, then blending those sounds to form words (e.g., reading unfamiliar words or nonsense words); Decoding Speed (DS), the ability to quickly and accurately translate written symbols (letters, letter patterns) into their corresponding sounds or words that involves the speed and efficiency of phonological and orthographic processing during word recognition tasks; Reading Fluency (RF), the rate at which connected text can be read, typically measured in words per minute, that reflects the reader’s fluency in processing continuous prose while maintaining comprehension; and Reading Comprehension (RC), the ability to understand, interpret, and derive meaning from written text that involves integrating word recognition with language.

In addition to the CHC categorization, subtests were also identified as “good” or “poor” indicators of broad and narrow CHC abilities. For this study, only those tests categorized as “good” were included in the analysis. Subtests were considered good indicators of a CHC ability based on the task demands and characteristics of the subtests, information from their respective technical manuals, and previous research that has been conducted with those subtests. Subtests that were considered “good” indicators were those that appeared to measure a single broad CHC ability via expert consensus and did not include task demands and characteristics that may confound test scores. For example, a subtest like Verbal Analogies, designed to measure Fluid Reasoning [Gf], also measures Comprehension-Knowledge [Gc] to a substantial degree (e.g., LaForte et al. 2025). Excess reliable variance that is associated with other distinct constructs, known as construct-irrelevant variance (Messick 1989), complicates interpretation. Generally, a subtest with a moderate to strong loading on one factor and insignificant loadings on all others is considered a relatively pure (i.e., good) measure of an ability (Woodcock 1990). When subtests appeared to primarily measure a single CHC ability, based on converging data sources (e.g., expert consensus and previous factor analytic studies), they were categorized as good. In contrast, those that tended to have cross-loadings or inconsistent findings were categorized as poor indicators.

Examples of good and poor indicator categorizations are provided. Well-established subtests, such as Vocabulary and Information from the Wechsler family of tests, were categorized as “good” indicators of a broad CHC ability because ample evidence shows they primarily measure one broad ability, Gc. Conversely, the Arithmetic subtest, also from the Wechsler family of tests, was categorized as a “poor” indicator. This is because the test requires examinees to listen to a mathematics word problem and solve it without using pencil and paper. This subtest requires several cognitive processes, including Auditory Working Memory (i.e., Gwm), Quantitative and Sequential Reasoning (i.e., Gf), and Listening Comprehension (i.e., Gc). Previous research has suggested that Arithmetic is a mixed measure of abilities, loading on Gf, Gwm, and Gc factors across different Wechsler batteries and in independent joint factor analytic studies (e.g., Flanagan et al. 2013; Niileksela and Reynolds 2019). Careful attention was paid to the categorization of subtests, ensuring that only those minimally affected by construct-irrelevant variance were included.

### 2.4. Step 1: Estimating Meta-Analytic Correlations

The *metafor* package for R was used to estimate all meta-analytic correlations (Viechtbauer 2010). A three-level random effects model was used when estimating the meta-analytic correlations. In this model, the level 1 random effect is the within-study sampling error, or the sampling error for each correlation included in the meta-analysis. This is used to weight the correlations when estimating the meta-analytic average, where more precise estimates (i.e., those with larger sample sizes) receive greater weight. The level 2 random effect is the variance between studies included in the meta-analytic correlation, or the distribution of correlations around the meta-analytic average of the correlations. The level 3 effect accounts for variance for correlations that are clustered within the same study or in the same correlation matrix for this study. For example, when estimating the meta-analytic correlation between Gc and Gf, there were over 1800 correlations from around 200 different correlation matrices; thus, many of the correlations used to calculate the meta-analytic average correlation between Gc and Gf were derived from the same correlation matrix and the same sample. The third level of the model provides variance estimates of correlations within those matrices. In the output, each meta-analytic correlation has an average, a standard error (level 1); the variance around the meta-analytic average correlation, which represents the random effect (level 2, the assumption being that there is not one single correlation between Gc and Gf, but a distribution of values around that average correlation); and the variance due to the clustering of multiple correlations that measure Gc and Gf in the same correlation matrix (level 3, where correlations within the same correlation matrix are clustered because they are estimated from the same people).

All correlations were converted using Fisher’s *z*-transformation before analysis, and then the meta-analytic correlations and confidence intervals were back-transformed to Pearson’s *r* for reporting. Correlations were weighted using the inverse variance method (i.e., reciprocal of the squared standard error estimate for Fisher-transformed correlations, or $1/\sqrt{{\left(N-3\right)}^{2}}$). The restricted maximum likelihood estimator (MLE) was used to estimate all parameters. The restricted MLE was used because it provides unbiased variance estimates when estimating variance components (i.e., level 2 and 3 effects) and is particularly suitable for smaller sample sizes, and it is very similar to standard MLE with large sample sizes.

In addition to the meta-analytic correlations among broad cognitive abilities and reading skills, the between-variance (i.e., level 2) and within-variance (i.e., level 3) components are also reported. The Q and I^2^ statistics were also calculated to evaluate heterogeneity among correlations. Q is a test of overall heterogeneity (i.e., whether observed variation in correlations would be expected by chance alone), and the I^2^ statistic represents the percentage of variation in correlations across studies due to true heterogeneity rather than sampling error alone. The I^2^ is calculated using the Q statistic, where I^2^ = (Q − df)/Q.

### 2.5. Stage 2: Using the Meta-Analytic Correlation Matrix to Examine Cognitive–Reading Relations

A structural equation model (SEM) was estimated using the meta-analytic correlation matrix for the broad cognitive abilities and narrow reading skills as input data. These models were estimated with *Mplus* 7.4 (Muthén and Muthén 1998–2017). The focus of these analyses is primarily on the size of the estimates rather than their statistical significance. For this analysis, standardized path coefficients were interpreted using Keith’s (2019) recommendations, where coefficients < 0.05 were considered negligible, coefficients between 0.05 and 0.09 were considered small, coefficients between 0.10 and 0.24 were considered moderate, and coefficients > 0.24 were considered large. The sample size for the SEM can be set in different ways, including the sum of all individuals included across all samples used, the median of sample sizes across correlation matrices, or the harmonic mean across correlation matrices (Cheung 2015; Viswesvaran and Ones 1995). Due to the large number of meta-analytic correlations estimated in this study and the large number of correlation matrices included, a combination of these approaches was used. The total sample size used to estimate each correlation pair was calculated first, and then the median of those sample sizes was used as the overall sample size when calculating the SEMs. The median sample size across all meta-analytic correlation pairs was 1764 (range = 92–2901). This was used as the sample size when estimating the SEM. Note that the sample size does not affect the magnitude of the parameter estimates in the model (e.g., size of factor loadings and regression coefficients), but it does affect the standard errors for those parameter estimates. Because the interpretation of the results is primarily focused on the size of the structural paths, their statistical significance was of secondary importance.

An integrated achievement model was estimated where foundational reading skills were included as predictors of advanced reading skills. Here, the broad cognitive abilities were included as predictors of all reading skills. Phonological Decoding was a predictor of Lexical Decoding, Decoding Speed, Reading Fluency, and Reading Comprehension. Lexical Decoding was a predictor of Decoding Speed, Reading Fluency, and Reading Comprehension. Decoding Speed was a predictor of Reading Fluency and Reading Comprehension, and the Reading Fluency was a predictor of Reading Comprehension. This model assumes that foundational reading skills are important precursors to advanced reading skills.

## 3. Results

### 3.1. Descriptive Statistics

The full dataset used for this study included 49,959 correlations across 599 different correlation matrices extracted from 137 different test batteries. Each correlation matrix included in the dataset for this analysis included an average of 83.40 correlations, ranging from 1 to 1227 correlations. On average, the sample size for each correlation matrix was 328.39, with a range of 18 to 2901. A total of 196,704 individuals were included across all correlation matrices analyzed for this study.

On average, 295.61 correlations were used to estimate each correlation between broad cognitive abilities and reading skills, with a range of 9 to 3197 correlations. These were derived from an average of 87 different correlation matrices, with a range from 14 to 378 different matrices. The total sample size included in the estimation of each correlation pair was, on average, 1772.43 people, with a range of 92 to 2901 people.

### 3.2. Meta-Analytic Correlations

**Figure 1:**
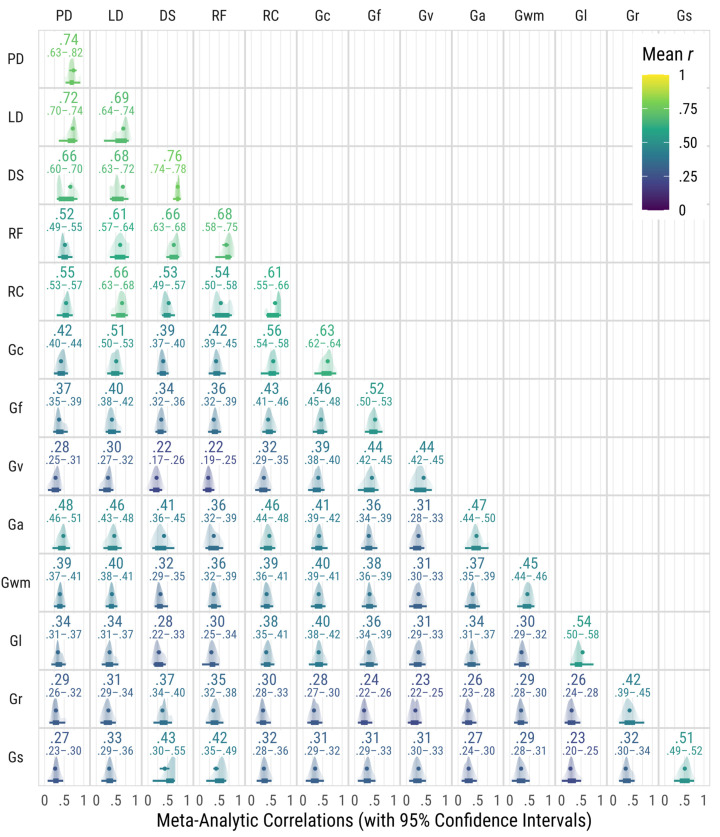
Meta-analytic correlations among broad CHC cognitive abilities and reading skills. The line below the density plot shows the middle 66% and 95% of the correlation distribution weighted by sample size. Gc = Comprehension-Knowledge, Gf = Fluid Reasoning, Gv = Visual Spatial Processing, Ga = Auditory Processing, Gl = Long-Term Storage, Gr = Retrieval Fluency, Gwm = Short-Term Working Memory, Gs = Processing Speed, LD = Lexical Decoding, PD = Phonological Decoding, DS = Decoding Speed, RF = Reading Fluency, RC = Reading Comprehension.

The number of correlations used to estimate meta-analytic correlations are in Table 1. The number of correlation matrices that included correlations used to estimate meta-analytic correlations are in Table 2. All meta-analytic correlations among broad cognitive abilities and reading skills are in Figure 1. All meta-analytic correlations were statistically significantly different from zero. The 95% confidence intervals for the correlations were relatively small, especially for those with a very large number of correlations (e.g., between Gc and Gwm).

For the broad cognitive abilities, the largest correlations were between tests that measure the same broad ability, providing some assurance that the categorization of tests under the different CHC abilities was appropriate. Only the correlation within Gv tests had the same correlation between Gv and Gf tests (0.44), which is not unexpected given that Gf tests often use visual stimuli, and some researchers have suggested that these abilities may represent a more general perceptual reasoning ability rather than separate Visual Spatial and Fluid Reasoning abilities (Grégoire 2017).

For the narrow reading skills, the largest correlations for each were between tests that measure the same narrow ability except for Lexical Decoding, where the largest correlation was with Phonological Decoding. Overall, reading skills correlated more highly with each other than the broad cognitive abilities, which is expected since all of these tests measure various reading skills. However, the correlations are high but not necessarily approaching perfect correlations, suggesting that they may be viewed as related but distinct skills.

When examining the correlations between broad cognitive abilities and reading skills, several observations are notable. For Decoding Speed, the largest correlation was with Gs followed by Gc. For Reading Fluency, the largest correlation was with Gc followed by Gs. Finally, for Reading Comprehension, the largest correlation was with Gc, which was not surprising given the language demands of Reading Comprehension tests. The next largest correlations were with Ga and Gf.

### 3.3. Heterogeneity of Meta-Analytic Correlations

**Figure 2:**
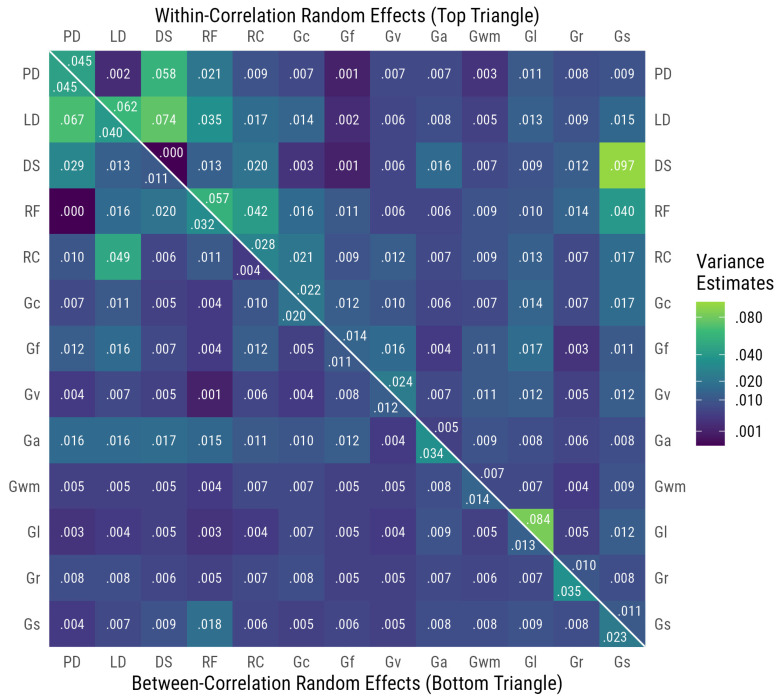
Variance estimates of between- and within-random effects. Ability abbreviations are the same as in Figure 1.

Figure 2 includes the level 2 variance estimates (below the diagonal) and level 3 variance estimates (above the diagonal), representing the variance for the between-level random effect and within-level random effect, respectively. For other indicators of heterogeneity, all *Q* values for meta-analytic correlations were statistically significant at the *p* < .001 level, suggesting substantial heterogeneity in the correlations used to estimate the meta-analytic correlation values. The *I*^2^ values were also large, with an average *I*^2^ value of 87.8% and a range between 73.8% and 97.1%, indicating that at least 73%, and in some cases, nearly all of the heterogeneity in the meta-analytic correlations is due to true heterogeneity rather than sampling error. This suggests that there may be important moderators of correlations that would be worth considering.

### 3.4. SEM Using the Meta-Analytic Correlation Matrix

**Figure 3:**
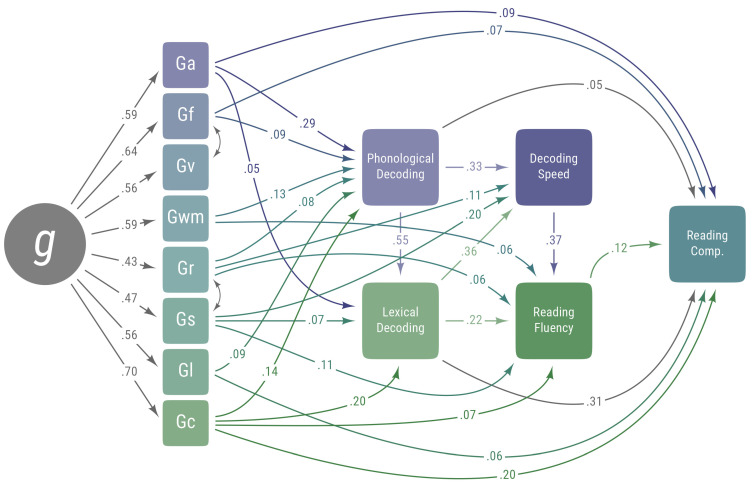
Integrated cognitive–reading structural equation model using meta-analytic correlation matrix. Standardized coefficients less than 0.05 were omitted. Abbreviations are the same as in Figure 1.

The meta-analytic correlation matrix in Figure 1 was used as input data for the SEM. To estimate the SEM, all values on the diagonal of this matrix were set to 1, and the means and standard deviations for each variable in the correlation matrix were set to 0 and 1, respectively. Model fit was excellent, χ^2^ (18) = 40.18, *p* = .002, CFI = 0.998, TLI = 0.990, RMSEA = 0.026, SRMR = 0.011. In this model, all estimates of Gv to reading skills were not statistically significant or were statistically significant and negative, suggesting Gv may act as a suppressor for some variables and may artificially inflate certain other path coefficients. The paths from Gv to reading skills were removed, and the model was reestimated. Although the Δχ^2^ between these models was statistically significant, Δχ^2^ (Δ*df* = 5) = 27.30, *p* < .001, the model fit was still excellent, χ^2^ (23) = 67.48, *p* < .001, CFI = 0.995, TLI = 0.984, RMSEA = 0.033, SRMR = 0.013. The integrated cognitive–reading SEM results using the meta-analytic correlation matrix is in Table 3. Figure 3 showcases the integrated cognitive–reading achievement meta-analytic SEM model.

The standardized factor loadings from *g* to the broad cognitive abilities were all moderate in size: Gc = 0.70, Gf = 0.64, Gv = 0.56, Ga = 0.59, Gl = 0.56, Gr = 0.43, Gwm = 0.59, and Gs = 0.47. It is important to note that all of the correlations in the meta-analysis were among single subtests, so these would not represent the factor loadings if composite scores or latent variables were used. However, the observation that Gc and Gf have the largest loadings and speeded tests of Gs and Gr have the lowest loadings is consistent with previous research (Carroll 1993).

In each of the following sections, the total effects of the broad cognitive abilities and reading skills are described. For Lexical Decoding, Decoding Speed, Reading Fluency, and Reading Comprehension, the total effects of broad abilities are highlighted and interpreted, even when the direct effects may be small. For Phonological Decoding, Ga had a large effect, Gc, Gl, and Gwm had moderate effects, Gf and Gr had small effects, and Gs had a negligible effect. The indirect effect of *g* was large, and the *R*^2^ was 0.34. Overall, this suggests that the ability to decode nonsense words is primarily predicted by auditory processing, but a range of different cognitive abilities contributed to the prediction of Phonological Decoding.

For Lexical Decoding, Gc and Phonological Decoding had large effects, Ga and Gwm had a moderate effect, and Gf, Gl, Gr, and Gs had small effects. The indirect effect of *g* on Lexical Decoding was large, and the *R*^2^ was 0.59. Overall, this suggests that the ability to decode words using phonic knowledge (and, in turn, Ga) and the ability to decode words using background knowledge were the most important predictors of decoding and recognizing real words.

For Decoding Speed, there were large total effects from Lexical Decoding, Phonological Decoding, and Gs. There were moderate total effects from Gc, Ga, and Gr and a small total effect from Gf. The total effects from Gl and Gwm were negligible. The indirect effect of *g* was large, and the *R*^2^ was 0.57. This suggests that the ability to decode words is highly predictive of decoding words quickly. Additionally, general cognitive processing speed also contributes to speeded decoding skills.

For Reading Fluency, the variables of Phonological Decoding, Lexical Decoding, and Decoding Speed all had large effects. There were moderate total effects from Gc, Ga, Gr, Gwm, Gs, and Phonological Decoding, a small total effect from Gf, and a negligible effect from Gl. The indirect effect of *g* was large and the *R*^2^ was 0.52. This suggests that the ability to read words is the strongest single predictor of a person’s ability to read connected text (i.e., sentences, paragraphs). Still, there are effects from a broad range of cognitive abilities.

For Reading Comprehension, there were large effects from Gc, Phonological Decoding, and Lexical Decoding. There were moderate effects from Gf, Ga, Gl, and Reading Fluency and small effects from Gr, Gwm, Gs, and Decoding Speed. The indirect effect from *g* was large, and the *R*^2^ was 0.55. The large effects from G*c* and Lexical Decoding suggest that the ability to decode words accurately and a person’s language skills are important predictors of their ability to understand what they read, which is consistent with the Simple View of Reading (Gough and Tunmer 1986).

## 4. Discussion

This study used meta-structural equation modeling to analyze integrated models of cognitive–reading relations using 599 different correlation matrices from 137 distinct cognitive and achievement test batteries that included over 49,000 correlations. The purpose of this study was to combine these data and develop a consensus integrated model of cognitive–reading relations that brings together much of the available data from standardized, norm-referenced cognitive and achievement tests. The results of this research largely support previous work on cognitive–reading relations and provide an important integration of data to help clarify and extend the understanding of cognitive–reading relations and theoretical models of reading.

### 4.1. Basic Reading

With regard to basic reading skills, Comprehension-Knowledge and Auditory Processing were primary contributors to Lexical Decoding. Auditory Processing exerted the largest effect on Phonological Decoding which had moderate associations with Comprehension-Knowledge, Long-Term Storage, and Short-Term Working Memory. These findings are largely consistent with previous work suggesting that both Gc and Ga are strong predictors of word decoding skills (e.g., Floyd et al. 2012; Niileksela et al. 2016). In this study, subtests that include real words were examined separately from nonsense words, and the results suggest that there are some differential relations between these two types of tasks, namely, that Ga was more strongly predictive of reading nonsense words.

### 4.2. Reading Fluency

The Direct and Indirect Effects Model of Reading (DIER; Kim 2023) asserts that word reading—and to a lesser extent, language comprehension—give rise to text fluency, all of which are supported by cognitive abilities. This study found that Phonological Decoding, Lexical Decoding, and Processing Speed contributed to Decoding Speed, which, in turn, played a large role in Reading Fluency. Moreover, Lexical Decoding, Comprehension-Knowledge, and Processing Speed also directly contributed to Reading Fluency. As highlighted in DIER and the Comprehensive Discourse Model of Comprehension (CMDC; Van Den Broek and Kendeou 2022), reading is neither a bottom-up nor a top-down process but rather multiple automatic and strategic processes occurring in different combinations throughout reading (Kintsch 2012). This may help explain why Lexical Decoding contributed to Reading Fluency both directly and via Decoding Speed. The role of Comprehension-Knowledge in Reading Fluency may be explained in a similar fashion. Although readers’ ability to retrieve known words from an “internal dictionary” (Lexical Decoding) may facilitate the speed at which they read, the dictionary is constructed, in large part, thanks to their background knowledge.

### 4.3. Reading Comprehension

Lexical Decoding and Comprehension-Knowledge exerted direct effects on Reading Comprehension. This may reflect the aforementioned internal dictionary that is constructed using cultural background knowledge and that readers draw on to recognize words for easy comprehension. According to CMDC, a given sentence triggers the activation of concepts in background knowledge, updating one’s “situation model” of the overall text. As previously noted, Long-Term Storage, Short-Term Working Memory, and Processing Speed helped predict decoding and fluency. However, not only did decoding and fluency directly impact Reading Comprehension—in line with DIER—but they also served as mediators of Comprehension-Knowledge and Auditory Processing. As specified by CMDC, the information immediately available to the reader—which draws from the spread of activation from earlier text and background knowledge—is constrained by cognitive resources (e.g., working memory) and lower-level processes (e.g., decoding; Van Den Broek and Espin 2012). Thus, cognitive abilities and basic reading skills may be potentially constraining the integration of background knowledge and capacity to update the situation model. Moreover, the direct effects of Fluid Reasoning and Long-Term Storage may reflect the use of reading strategies when lower-level skills or background knowledge fail to meet readers’ standards for coherence (Van Den Broek and Kendeou 2022). Furthermore, this pattern may help to explain some of the inconsistencies surrounding the roles of cognitive abilities in Reading Comprehension. For instance, some have argued that when other skills are accounted for, working memory is no longer directly related to Reading Comprehension (see Kim 2023). This study clears some of the empirical smoke, revealing that Short-Term Working Memory predicts basic skills like Phonological Decoding and Reading Fluency, which in turn predict Reading Comprehension.

### 4.4. Additional Findings

Visual Processing did not have a consistent relationship with reading skills, but all other CHC broad cognitive abilities had a relationship with reading skills. Retrieval Fluency (Gr) was previously viewed as part of Learning Efficiency (Gl) (formerly combined as Glr), but other evidence suggested Gr and Gl should not be grouped together under a single broad ability (Schneider and McGrew 2018). Because Gr and Gl are both speeded types of tests, some may consider them to be under a more general cognitive speed factor. Still, the observation that both Processing Speed (Gs) and Gr contribute unique variance to these reading outcomes suggests that they are different abilities that differentially predict outcomes (if they were the same, we would expect them to predict the same variance in an outcome).

### 4.5. Practical Implications

There are several important practical implications of this study, especially when considering the assessment of reading skills when there are concerns about reading development. First, Reading Comprehension was not solely explained by basic reading skills. Although Lexical Decoding and Reading Fluency played a vital role, Comprehension-Knowledge and Auditory Processing also had effects through their connections with lower-level skills. Such findings align with integrated cognitive–reading models, DIER, and CMDC and may warrant new frameworks for assessment and instruction. Traditionally, it was thought that if basic reading skills were compromised, readers would become hindered in their ability to extract meaning from text, suggesting that such skills (i.e., decoding) are a prerequisite for comprehension (Perfetti 1985). Since then, several studies have come into focus that suggest decoding and Reading Comprehension vary substantially in their correlations and develop simultaneously and independently rather than sequentially (Garcia and Cain 2014). The current findings add to this line of work by highlighting the mediating roles of cognitive abilities. Akin to reviews by Zaboski et al. (2018) and Bryan and Mayer (2020), Comprehension-Knowledge and Auditory Processing emerged as important for basic reading and Reading Comprehension. Also similar to previous work, Long-Term Storage and Short-Term Working Memory had moderate and small effects for basic and advanced reading. Departing from Zaboski et al. (2018), the current study found general intelligence to be relatively less influential for basic reading (0.35 vs. 0.74) and Reading Comprehension (0.55 vs. 0.76). However, it is important to note that the correlations used in the current study were at the subtest level, whereas the previous meta-analyses focused on relations at the composite or latent variable level.

The findings also warrant more dynamic and comprehensive assessment models for reading difficulties and disorders such as dyslexia. For instance, bifactor models of specific learning disorders (SLDs) propose that academic difficulties reflect a general SLD liability factor and domain-specific dimensions (Peterson et al. 2021). Assessment protocols should thus be structured to identify cross-cutting impairments and skill-specific deficits, for example, distinguishing a student with generalized processing limitations from one with isolated Phonological Decoding impairments. This supports a consensus model in which both latent-level commonality and surface-level heterogeneity are meaningfully captured, particularly when determining eligibility for services or tailoring intervention.

### 4.6. Limitations and Future Directions

The findings and subsequent inferences drawn from this study should be considered in light of several important limitations. The first limitation concerns sampling issues. Although we used data amassed from multiple datasets representing thousands of correlations, the information analyzed was drawn from extant standardization and special validity samples across a multitude of cognitive and achievement test batteries. These are large-scale datasets and samples that allow for strong statistical power that were intended to be representative of the U.S. population. However, these samples are not robust representations of special populations (e.g., dyslexia) and may not generalize to those with a variety of academic reading difficulties. Future research would benefit from the increased use of special validity samples and special populations (e.g., twice exceptionalities) to better understand the dynamic of meta-analytic correlations between cognitive abilities and reading difficulties. Similarly, reading achievement varies substantially across languages with different orthographic structures (e.g., Seidenberg 2013; Seymour et al. 2003), and it is unknown whether our model that was derived from English-language data is applicable to languages that present with more transparent or opaque orthographies. Therefore, it is possible that the specific English-language context may have influenced the results to some degree. Future research should conduct cross-linguistic validation of the model given that its application to other languages is unknown. Regarding the effects of general intelligence versus broad abilities, there may be unobserved age and ability-level differences given that emerging evidence has indicated such effects for certain test batteries (Hajovsky et al. 2025). However, future work should be carried out using cross-battery approaches to examine the generalization of such effects.

There are measurement limitations of this study that are worthy of mention. A key strength of this study is the results were generated using meta-SEM with a cross-battery approach. However, we did not conduct a moderator analysis to test how cognitive–reading achievement relations may vary across age, test battery family, or at the subtest level. Additionally, it would be challenging to incorporate other aspects of meta-analysis (e.g., forest plots) due to the very large number of correlations used to estimate each correlation pair. Another limitation of this study is the use of cross-sectional data and a lack of consideration for developmental changes in reading skills. There are independent contributions to reading that shift across development (e.g., Catts et al. 2005; Foorman et al. 2018), and our models may not have captured the dynamic interplay of these skills that may be more fluid and in motion across development. Thus, our findings may be more specific to a particular developmental stage, and more research is needed to consider the dynamic interplay of cognitive abilities and reading skills over time. We made specific assumptions of the models. Empirical findings and assertions regarding the influence of one variable on another rest on the validity of the implied model. Our meta-analytic findings are based on the analysis of variable effects within a theoretically defined model. Furthermore, the use of integrated models assumes the effects of cognitive abilities on Reading Comprehension are partially mediated through basic reading skills (Hajovsky et al. 2014; Niileksela et al. 2016). We were unable to disentangle the presumed variable mediation and had to assume the mediation occurs instantaneously. Future research should leverage longitudinal data when addressing aims specifically focused on statistical mediation.

## 5. Conclusions

Cognitive abilities remain steadfast predictors of the acquisition and development of reading skills. In this landmark study, which meta-analyzed thousands of correlations across various cognitive and achievement test batteries, we demonstrated that broad cognitive abilities (e.g., Comprehension-Knowledge, Auditory Processing) are robust predictors of basic reading skills and Reading Comprehension and that basic reading skills are predictors of advanced reading skills. The development of a consensus model of integrated cognitive–reading achievement relations enriches our theoretical understanding of how cognitive abilities influence reading development and highlights that these construct relations are generalizable across a large sample of cognitive and achievement test batteries. The use of meta-structural equation modeling in cognitive–achievement relations research should continue to expand to other domains of achievement.

## Figures and Tables

**Table 1:** Number of correlations used to estimate meta-analytic correlations.

	Gc	Gf	Gv	Ga	Gl	Gr	Gwm	Gs	LD	PD	DS	RF	**RC**
Gc	3197												
Gf	1831	400											
Gv	3170	1219	1125										
Ga	852	386	400	190									
Gl	1257	679	1791	384	495								
Gr	1057	519	541	381	496	331							
Gwm	3778	1582	2756	566	1350	817	1619						
Gs	2215	824	1427	342	556	503	1929	501					
LD	625	197	206	215	175	236	322	169	62				
PD	386	124	128	136	109	153	181	113	155	9			
DS	250	76	56	125	48	203	132	56	99	67	27		
RF	283	93	87	103	80	166	129	91	113	71	89	26	
RC	544	153	160	159	135	213	236	145	224	129	84	115	25

Note. Gc = Comprehension-Knowledge, Gf = Fluid Reasoning, Gv = Visual Spatial Processing, Ga = Auditory Processing, Gl = Long-Term Storage, Gr = Retrieval Fluency, Gwm = Short-Term Working Memory, Gs = Processing Speed, LD = Lexical Decoding, PD = Phonological Decoding, DS = Decoding Speed, RF = Reading Fluency, RC = Reading Comprehension.

**Table 2:** Number of correlation matrices that included correlations used to estimate meta-analytic correlations.

	Gc	Gf	Gv	Ga	Gl	Gr	Gwm	Gs	LD	PD	DS	RF	RC
Gc	384												
Gf	216	150											
Gv	273	222	263										
Ga	82	49	55	34									
Gl	123	111	160	48	126								
Gr	112	89	107	65	70	76							
Gwm	252	199	278	63	142	106	220						
Gs	179	154	198	44	70	85	182	133					
LD	152	59	67	72	56	69	74	49	41				
PD	96	46	45	59	39	56	52	42	102	9			
DS	41	19	18	38	14	47	26	16	46	41	21		
RS	71	27	26	43	21	60	29	26	77	66	54	14	
RC	140	52	52	62	45	65	58	47	136	99	40	75	21

Note. Gc = Comprehension-Knowledge, Gf = Fluid Reasoning, Gv = Visual Spatial Processing, Ga = Auditory Processing, Gl = Long-Term Storage, Gr = Retrieval Fluency, Gwm = Short-Term Working Memory, Gs = Processing Speed, LD = Lexical Decoding, PD = Phonological Decoding, DS = Decoding Speed, RF = Reading Fluency, RC = Reading Comprehension.

**Table 3:** Integrated cognitive–reading structural equation model using meta-analytic correlation matrix.

	Phonological Decoding Direct (Indirect, Total)	Lexical Decoding Direct (Indirect, Total)	Decoding Speed Direct (Indirect, Total)	Reading Fluency Direct (Indirect, Total)	Reading Comprehension Direct (Indirect, Total)
**Gc**	**0.14**	**0.20** **(0.08, 0.27)**	**−0.02** **(0.14, 0.12)**	**0.07** **(0.11, 0.18)**	**0.20** **(0.12, 0.32)**
**Gf**	**0.09**	**0.04** **(0.05, 0.09)**	**0.01** **(0.06, 0.07)**	**0.03** **(0.05, 0.08)**	**0.07** **(0.04, 0.12)**
**Ga**	**0.29**	**0.05** **(0.16, 0.21)**	**0.03** **(0.17, 0.20)**	**−0.01** **(0.12, 0.11)**	**0.09** **(0.10, 0.19)**
**Gl**	**0.09**	**0.00** **(0.05, 0.05)**	−0.02 (0.05, 0.02)	**0.02** **(0.02, 0.04)**	**0.06** **(0.03, 0.09)**
**Gr**	**0.09**	**0.04** **(0.05, 0.09)**	**0.11** **(0.06, 0.17)**	**0.06** **(0.08, 0.14)**	**0.01** **(0.06, 0.07)**
**Gwm**	**0.13**	**0.04** **(0.07, 0.11)**	**−0.04** **(0.08, 0.05)**	**0.06** **(0.04, 0.10)**	**0.03** **(0.06, 0.08)**
**Gs**	0.03	**0.07** **(0.02, 0.09)**	**0.20** **(0.04, 0.25)**	**0.11** **(0.11, 0.23)**	**0.00** **(0.07, 0.07)**
**Phonological Decoding**	**–**	**0.55**	**0.33** **(0.20, 0.53)**	**0.01** **(0.32, 0.33)**	**0.05** **(0.23, 0.28)**
**Lexical Decoding**	**–**	**–**	**0.36**	**0.22** **(0.13, 0.36)**	**0.31** **(0.06, 0.37)**
**Decoding Speed**	**–**	**–**	**–**	**0.37**	**0.04** **(0.05, 0.08)**
**Reading Fluency**	**–**	**–**	**–**	**–**	**0.12**
**Indirect Effect of g**	**0.50**	**0.54**	**0.47**	**0.48**	**0.57**
**R^2^**	**0.34**	**0.59**	**0.57**	**0.52**	**0.54**

*Note:* Bold cells shaded darkest have large total effect, bold and italicized cells with moderate shading have moderate effects, cells with italicized text and lightest shading have small effects, cells with italicized text with no shading have negligible effects. Gc = Comprehension-Knowledge, Gf = Fluid Reasoning, Ga = Auditory Processing, Gl = Long-Term Storage, Gr = Retrieval Fluency, Gwm = Short-Term Working Memory, Gs = Processing Speed.

## Data Availability

The data are not publicly available but are located within publisher test manuals available to those who have permission to administer such tests.

## Supplementary Materials

The following supporting information can be viewed at: Table S1: Test batteries; https://drive.google.com/file/d/1Fid2iTeSCZc8bUQXyqkieWKtdcCBjQ_5/view?usp=drive_link (accessed on 1 June 2025), Table S2: Correlation matrices; https://drive.google.com/file/d/1RJ0OQpRQVZeAzs-fwqFaD-0S1jfccWSJ/view?usp=drive_link (accessed on 1 June 2025), Table S3: Subtest categorizations; https://drive.google.com/file/d/1D9f75YhTAip0ykGiMA-zoRosuogo0f6K/view?usp=drive_link (accessed on 1 June 2025), Table S4: Correlations; https://drive.google.com/file/d/1gITmG3b5Y9qSwGMwyN9RJ8PlkKEzOWpj/view?usp=drive_link (accessed on 1 June 2025).
